# Mothercraft and Child-Welfare Centres

**Published:** 1916-01-15

**Authors:** 


### MOTHERCRAFT AND CHILD-WELFARE CENTRES.*

### II. Difficulties and Problems.

Cookery classes are obviously more difficult to organise than those referred to in our previous article (see The Hospital of October 9, 1915, p. 37). It is important that individual members of the class should be able to take part in the actual culinary manipulations, but the equipment provided must be as nearly as possible of the kind that is available in their homes. If gas-stoves are not used in their tenements or cottages it is useless to work with these. Small oil-stoves or even a gas-ring attached to a burner have been usefully employed, and in a few places it may be possible to hold a class in a cottage, and to do the cooking at an ordinary range. By the St. Pancras School for Mothers arrangements have been made to give instruction in the women's homes, but this is not easy to organise.

It is apparently rather difficult to persuade mothers to attend cookery classes; they seem loth to admit that they have anything to learn in the way of cooking. Whatever the reasons, this difficulty is unfortunate, as such teaching is probably more necessary to them than any other, and if successfully followed, would have a marked effect on the nutrition of the children. The lessons may last about two hours, and the number in the class should be about eight to twelve. If a syllabus is drawn up, it should be an elastic one; it is suggested that the teacher may find it best to use the cheapest nutritious food available on the day of the lesson and to teach the class how best to utilise it. Examples cited refer to lessons in which three ways of cooking herrings were taught and where cod's head was served in three different ways. Popular dishes are onion dumplings, tomatoes and macaroni, various cheese dishes, suet puddings, and different kinds of bread. The women should always be supplied with full notes of the ingredients and their exact use, for they cannot be expected to take proper notes for themselves. It is important that the mothers should be allowed to taste what is made, and they should be encouraged to practise at home during the week the cooking of a dish demonstrated, and then, at a subsequent lesson at the school, be allowed to make the same dish. In every case the cost should be made clear. Sometimes during an interval when a dish is cooking, and nothing else can conveniently be begun, it is advisable to take the opportunity to give a short talk on food values or practical housekeeping.

### Advanced Classes.

The Board of Education memorandum also considers the question of more advanced classes which might be attended by the wives of better-class artisans or clerks, who can profit by more formal instruction on the lines of that offered by certain evening classes. Such persons do not require to be taught ordinary sewing, but they will appreciate a dressmaking or tailoring class where they can learn to cut out and to make garments for their children and for themselves. Lectures on home nursing, first aid, and hygiene may be given; the value of fresh air may be made an introduction to the causes and prevention of consumption, and the concerted measures now officially being undertaken may be explained and discussed. Then, again, the work of the medical inspection of school children is a subject which will interest mothers, who often do not realise the scope and aims of this work. Special points worth discussing are, dental hygiene, mouth breathing, tonsils and adenoids, rheumatism, and the common infectious fevers. Particularly important is the subject of measles, its dangers and remoter effects.

At all schools for mothers where classes are held in the afternoons there will have to be met the problem of what is to be done with the babies and toddlers brought by the mothers. The best solution is probably to provide a separate room in which a few cots and some toys are available. The little children are looked after and amused by voluntary helpers, while the talk or lesson is being given. It is always difficult to persuade mothers to part from their children, and the latter also resent separation; it is important that the mothers should be allowed to go freely to their children when they wish to do so. Failing a separate room, a corner can be kept free for the children and baskets or simple cots provided for the babies, so that the mothers' hands may be free.

### The Teachers.

No definite requirements as to the qualifications of the teachers have been suggested by the Board of Education, but it is obvious that simple teaching of the kind required in schools for mothers is more difficult to give effectively than more advanced instruction. The superintendent, who is personally familiar with the mothers and their homes, is the best person to give the health talks, with an occasional informal address by the doctor. Instruction in sewing should be in charge of someone who has professional knowledge of the subject; a dressmaker, or an ex-elementary school teacher may be found to volunteer. Cookery requires a trained teacher, but she should have some personal knowledge of the women's homes.

The local education authority is often willing to provide teachers. These are generally members of the evening school staff, who undertake such subjects as infant care, nursing, sewing, and cookery. There is, however, the danger that the syllabuses adopted will be more adapted for use in evening schools than for the type of class held at schools for mothers. Local authorities should allow their teachers considerable latitude in this matter and

* The previous article of this series appeared on October 9, 1915.

shorter lessons are called for. The London County Council supplies more teachers for schools for mothers than any other authority and it has already taken steps to meet the new regulations. The proposed arrangements will ensure effective and appropriate instruction on terms financially advantageous to schools for mothers.

In fine, the new class of institutions for which the Board of Education and the Local Government Board are now laying down conditions precedent to the reception of a grant, deal with more elementary matters than most. As an offshoot of the great institutions, however, the hospital manager, like the hospital almoner, keeps an eye upon them, realising the potential importance of what may be called for the moment the minor activities of institutional life.

### What to Read.

There is already available a considerable selection of small books on the subject of mothercraft, published by The Scientific Press, Southampton Street, Strand, and other houses, and the best of these have been noticed in our columns. There is every likelihood of a further increase in the supply of elementary text-books of this description, as well as of manuals for the assistance of those who will be called upon to give instruction at schools for mothers and infant consultations; for, as may be readily guessed from the encouragement now given by the Board of Education and the Local Government Board, such schools and classes, as rapidly as circumstances permit, are being established all over the country.

One of the latest of these manuals for mothers hails from the United States, and is entitled "What Every Mother Should Know."* Owing to the fact that the book is in many respects hardly adapted for the use of English mothers, a passing notice only is called for. The author, Dr. C. G. Kerley, is professor of diseases of children at the New York Polyclinic. The book has the advantage of being written in the simplest terms, and its direct and dogmatic statements will be readily comprehended by the average mother. Babyhood receives, naturally, the greatest amount of attention, but the scope of the book includes children up to six years of age. The instructions given are sound, but as they are adapted for American mothers, the American climate and diet they do not call for detailed mention or criticism in this country. The plan of the book is, however, good.

* New York: Paul B. Hoeber. 1915. Price 1s. 6d.

